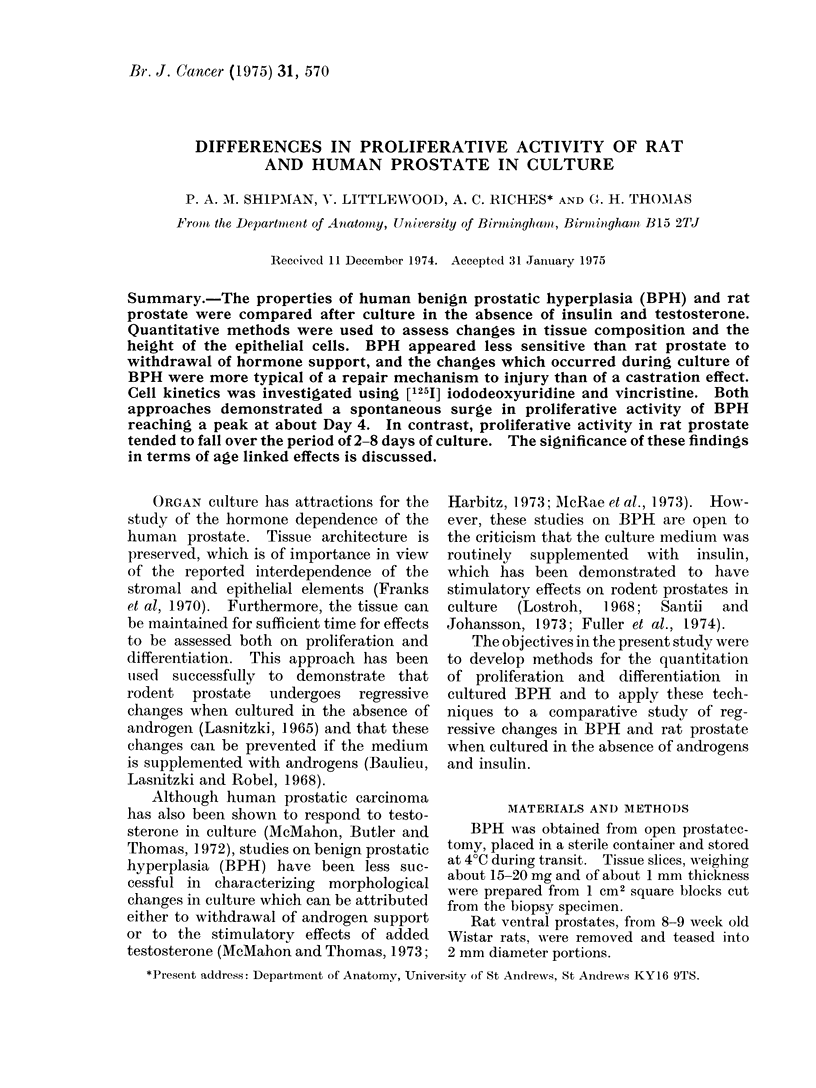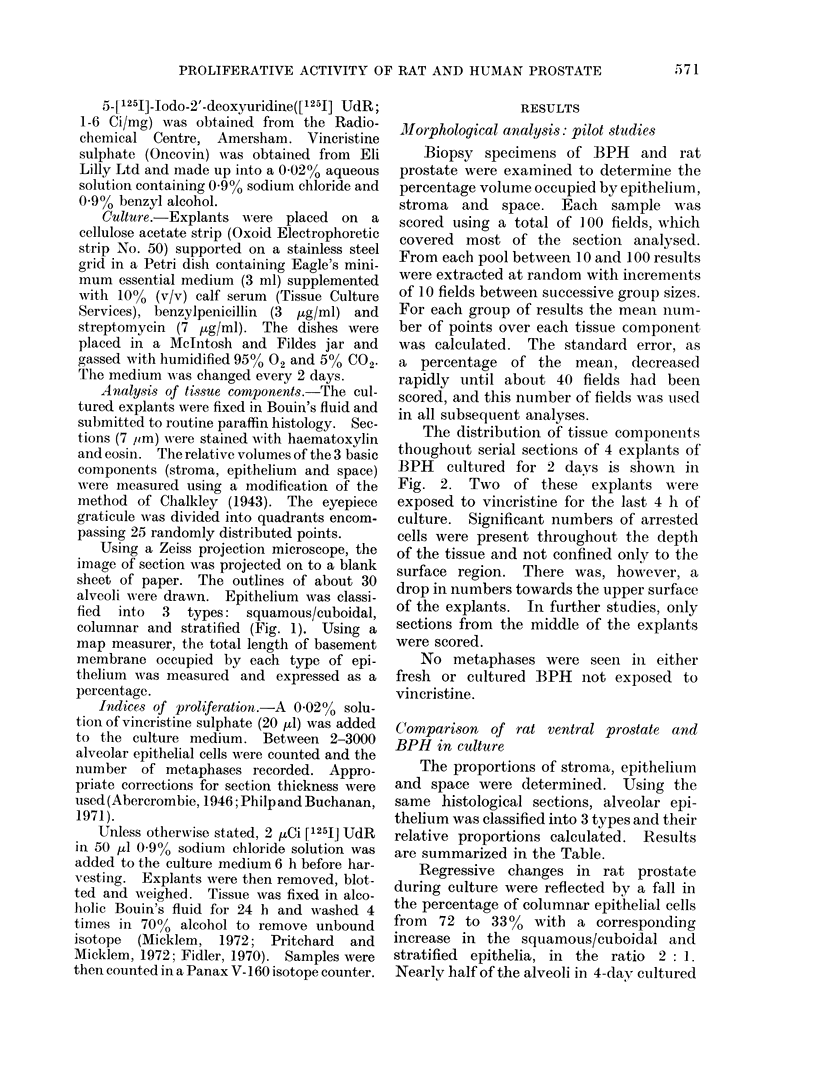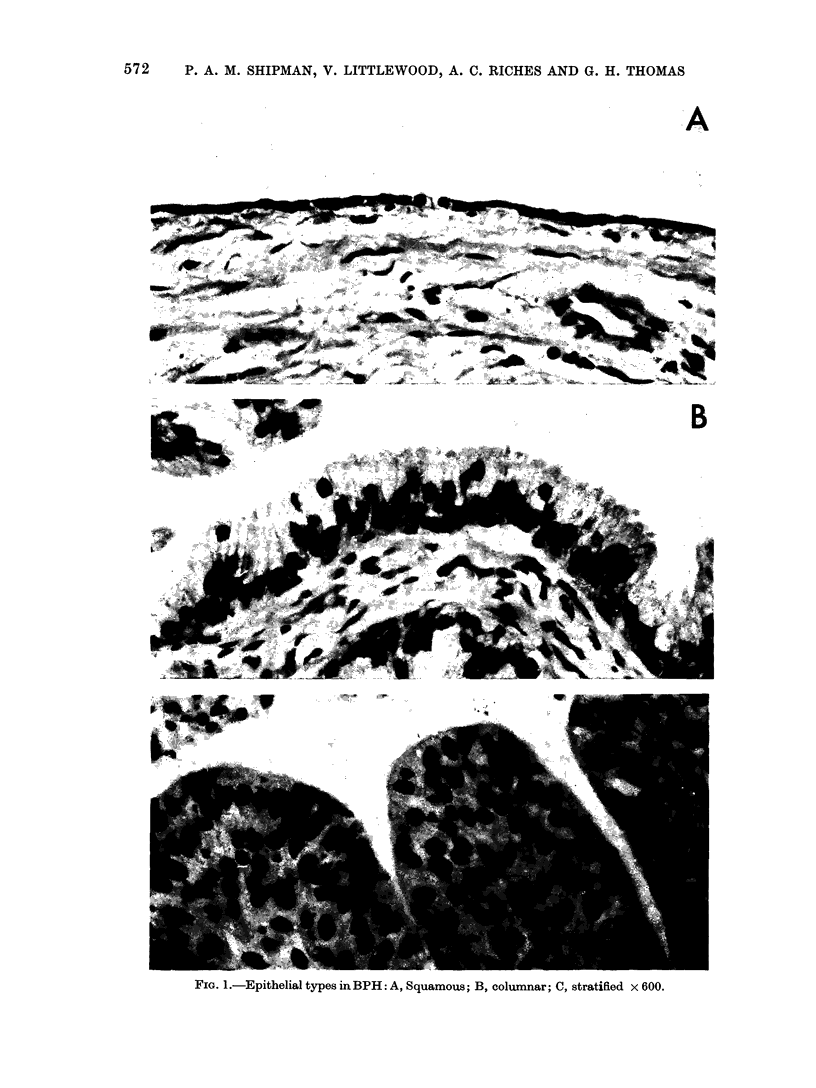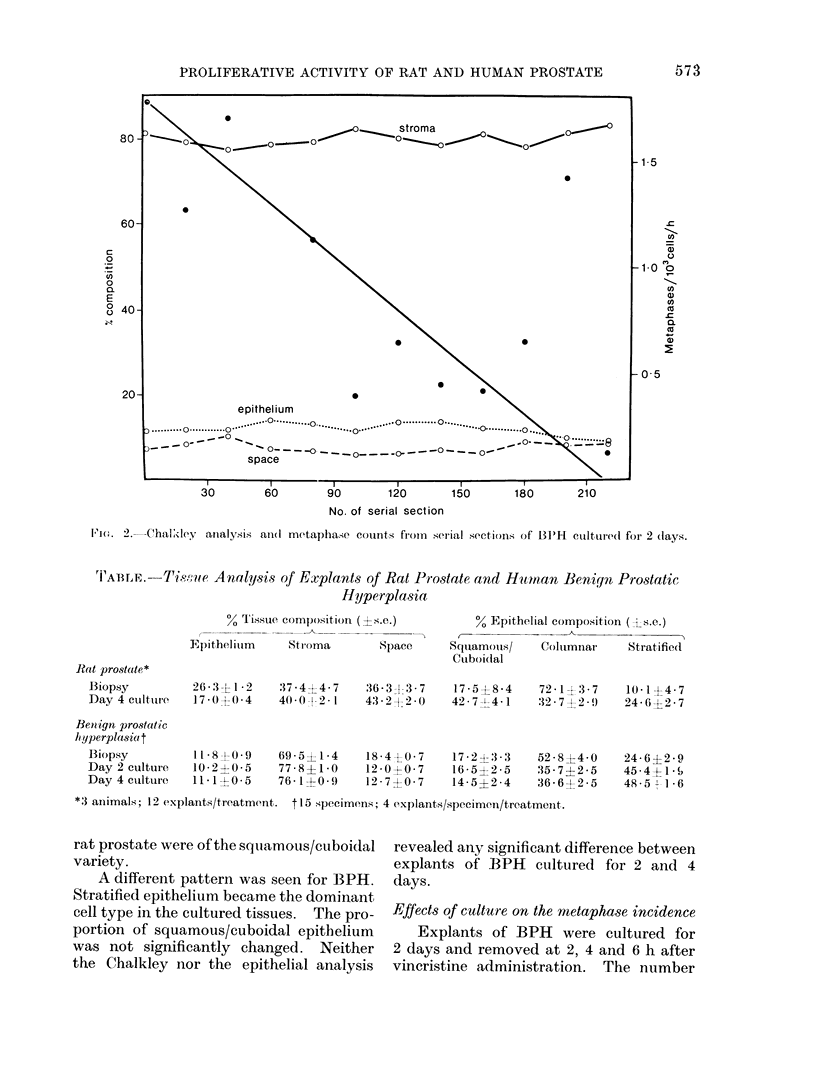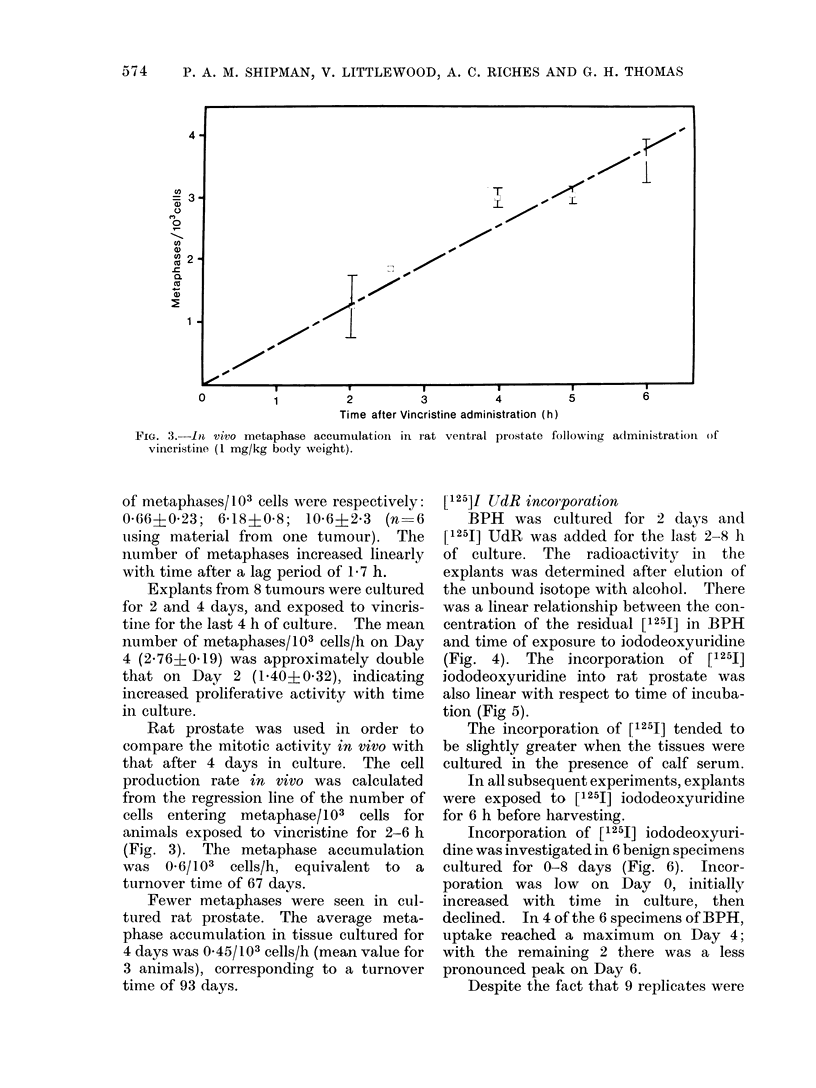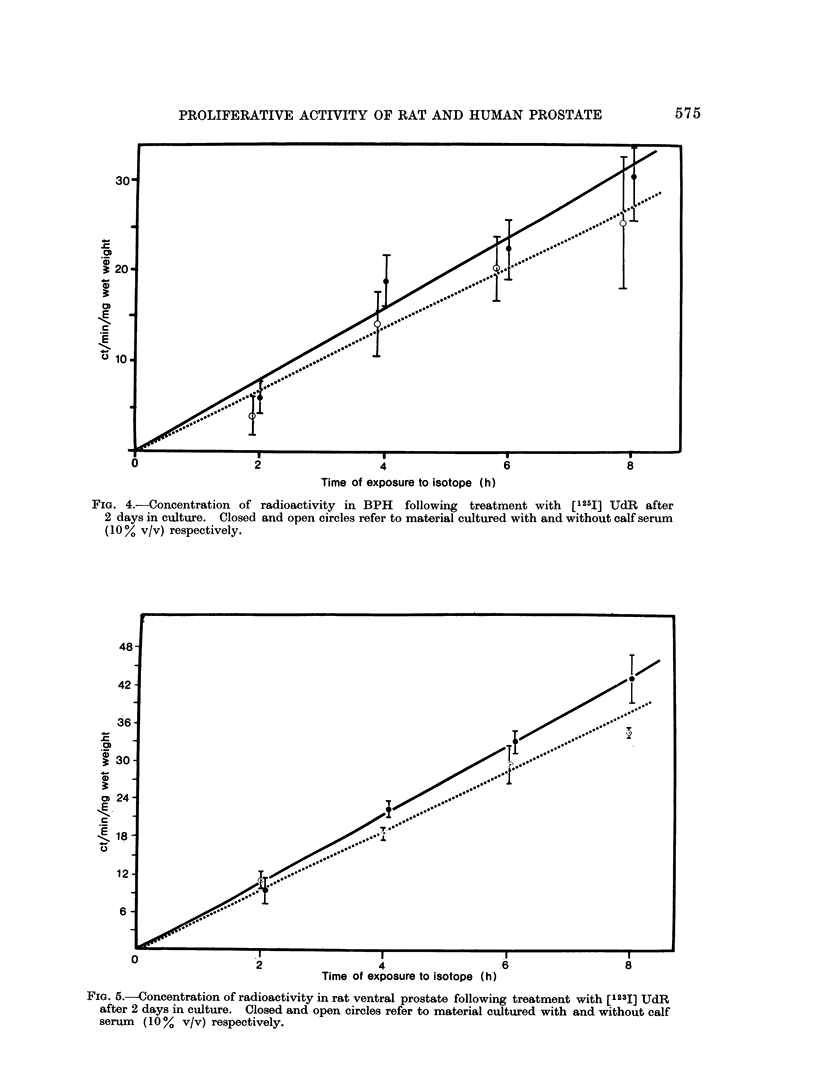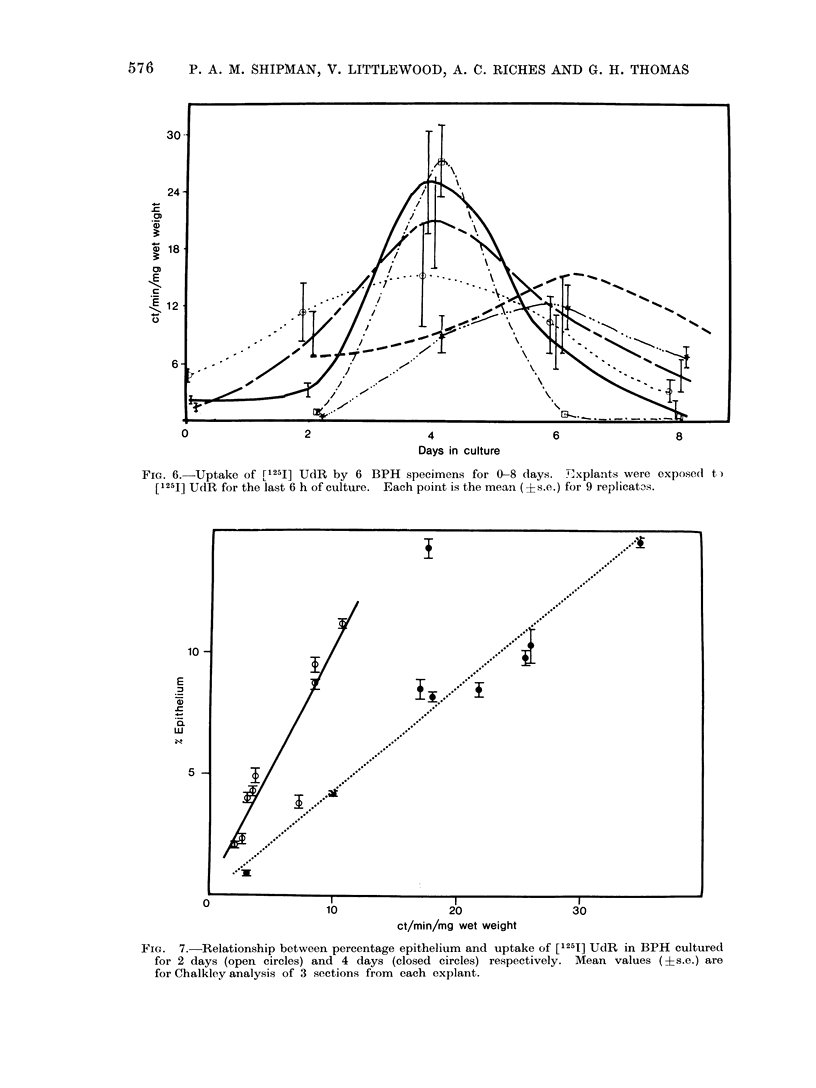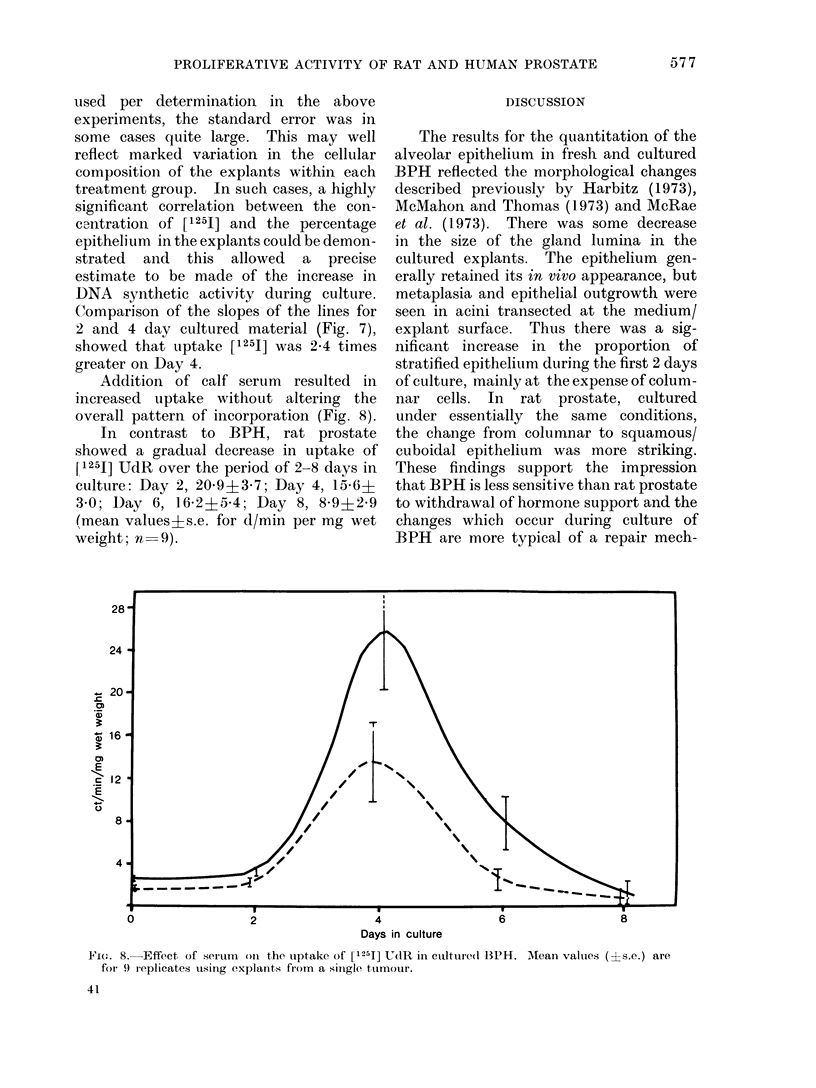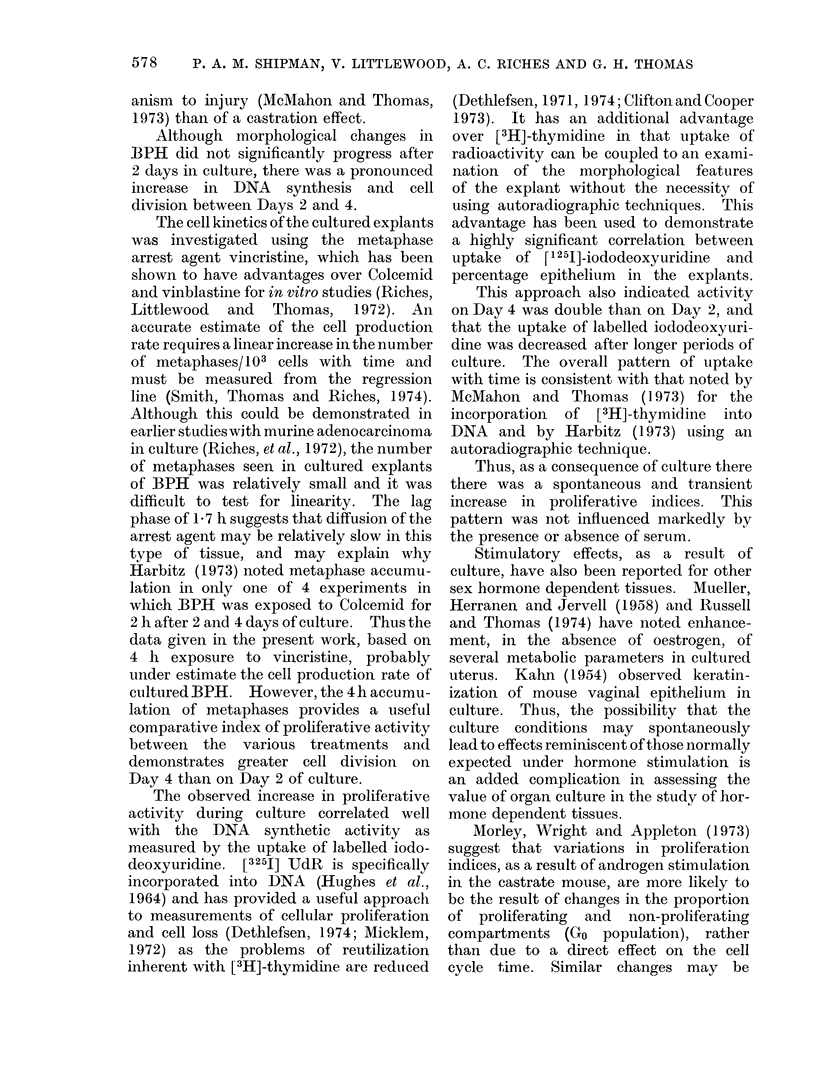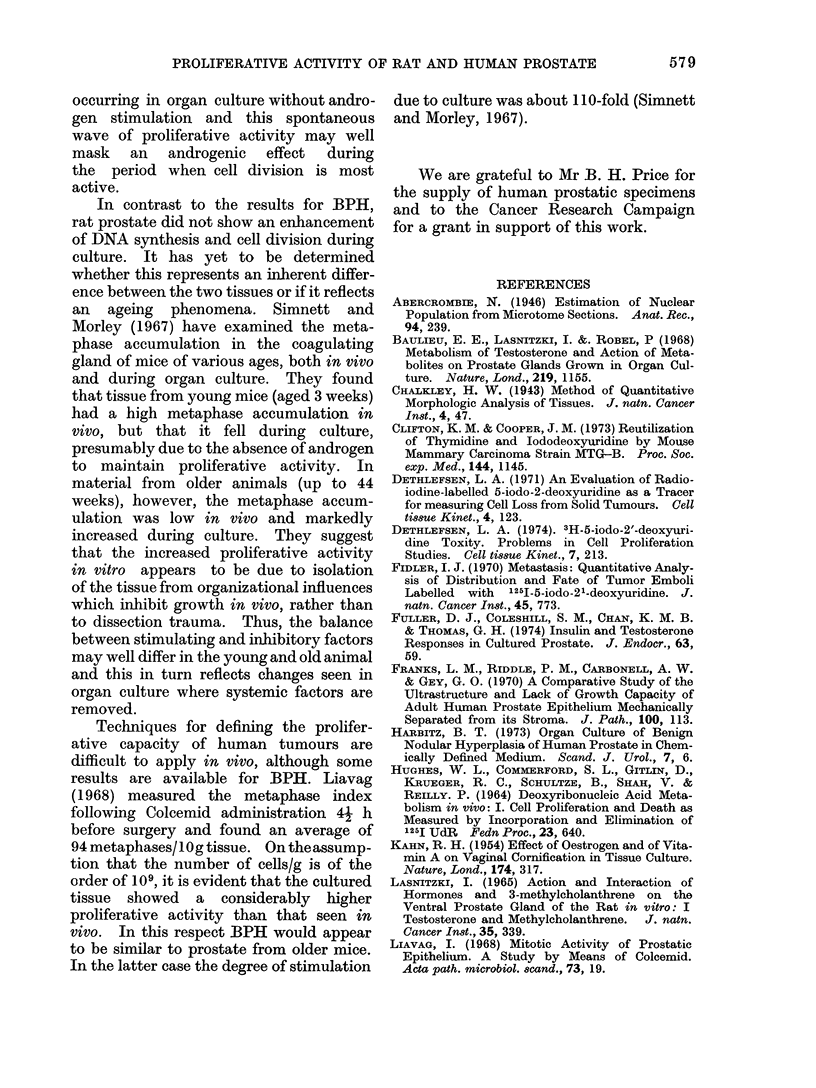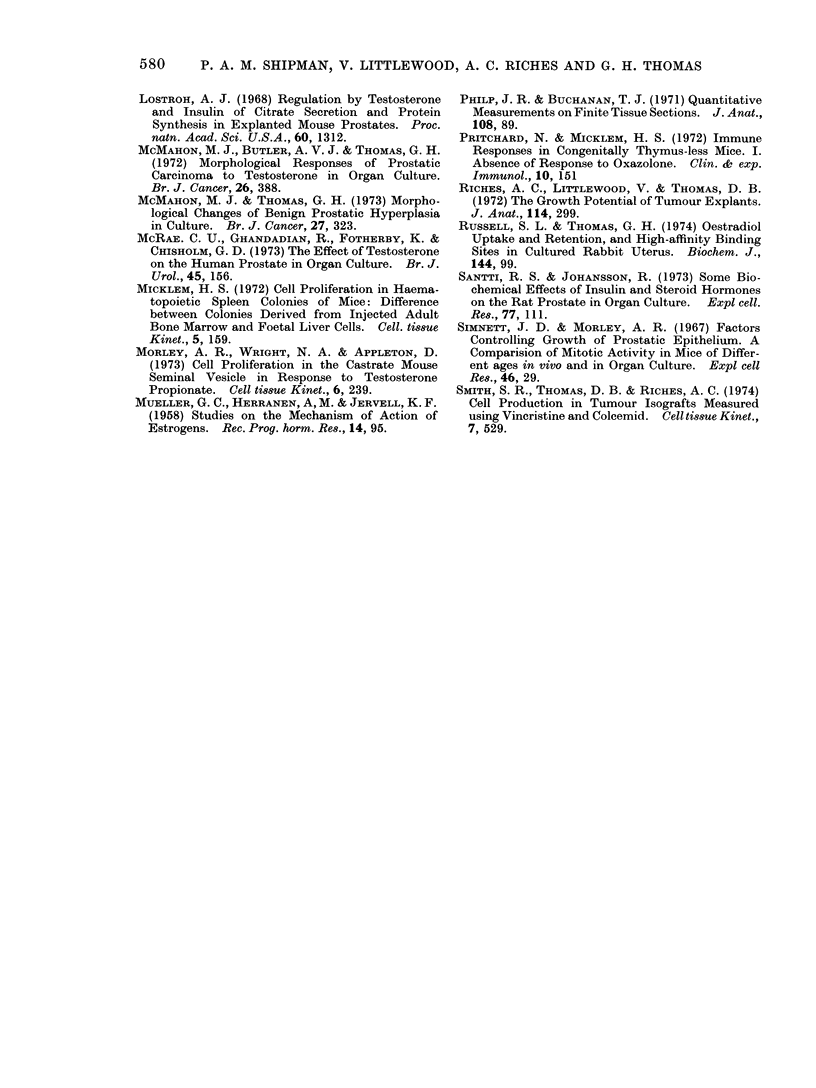# Differences in proliferative activity of rat and human prostate in culture.

**DOI:** 10.1038/bjc.1975.98

**Published:** 1975-05

**Authors:** P. A. Shipman, V. Littlewood, A. C. Riches, G. H. Thomas

## Abstract

**Images:**


*Br. J. Cancer* (1975) **31**, 570

### DIFFERENCES IN PROLIFERATIVE ACTIVITY OF RAT AND HUMAN PROSTATE IN CULTURE

P. A. M. SHIPMAN, V. LITTLEWOOD, A. C. RICHES* AND G. H. THOMAS

*From the Department of Anatomy, University of Birmingham, Birmingham B15 2TJ*

Received 11 December 1974. Accepted 31 January 1975

**Summary.—The properties of human benign prostatic hyperplasia (BPH) and rat prostate were compared after culture in the absence of insulin and testosterone. Quantitative methods were used to assess changes in tissue composition and the height of the epithelial cells. BPH appeared less sensitive than rat prostate to withdrawal of hormone support, and the changes which occurred during culture of BPH were more typical of a repair mechanism to injury than of a castration effect. Cell kinetics was investigated using [$^{125}$I] iododeoxyuridine and vincristine. Both approaches demonstrated a spontaneous surge in proliferative activity of BPH reaching a peak at about Day 4. In contrast, proliferative activity in rat prostate tended to fall over the period of 2–8 days of culture. The significance of these findings in terms of age linked effects is discussed.**

ORGAN culture has attractions for the study of the hormone dependence of the human prostate. Tissue architecture is preserved, which is of importance in view of the reported interdependence of the stromal and epithelial elements (Franks *et al*, 1970). Furthermore, the tissue can be maintained for sufficient time for effects to be assessed both on proliferation and differentiation. This approach has been used successfully to demonstrate that rodent prostate undergoes regressive changes when cultured in the absence of androgen (Lasnitzki, 1965) and that these changes can be prevented if the medium is supplemented with androgens (Baulieu, Lasnitzki and Robel, 1968).

Although human prostatic carcinoma has also been shown to respond to testosterone in culture (McMahon, Butler and Thomas, 1972), studies on benign prostatic hyperplasia (BPH) have been less successful in characterizing morphological changes in culture which can be attributed either to withdrawal of androgen support or to the stimulatory effects of added testosterone (McMahon and Thomas, 1973; Harbitz, 1973; McRae *et al.*, 1973). However, these studies on BPH are open to the criticism that the culture medium was routinely supplemented with insulin, which has been demonstrated to have stimulatory effects on rodent prostates in culture (Lostroh, 1968; Santii and Johansson, 1973; Fuller *et al.*, 1974).

The objectives in the present study were to develop methods for the quantitation of proliferation and differentiation in cultured BPH and to apply these techniques to a comparative study of regressive changes in BPH and rat prostate when cultured in the absence of androgens and insulin.

### MATERIALS AND METHODS

BPH was obtained from open prostatectomy, placed in a sterile container and stored at 4°C during transit. Tissue slices, weighing about 15–20 mg and of about 1 mm thickness were prepared from 1 cm$^2$ square blocks cut from the biopsy specimen.

Rat ventral prostates, from 8–9 week old Wistar rats, were removed and teased into 2 mm diameter portions.

*Present address: Department of Anatomy, University of St Andrews, St Andrews KY16 9TS.

5-[$^{125}$I]-Iodo-2′-deoxyuridine([$^{125}$I] UdR; 1·6 Ci/mg) was obtained from the Radiochemical Centre, Amersham. Vincristine sulphate (Oncovin) was obtained from Eli Lilly Ltd and made up into a 0·02% aqueous solution containing 0·9% sodium chloride and 0·9% benzyl alcohol.

*Culture.*—Explants were placed on a cellulose acetate strip (Oxoid Electrophoretic strip No. 50) supported on a stainless steel grid in a Petri dish containing Eagle's minimum essential medium (3 ml) supplemented with 10% (v/v) calf serum (Tissue Culture Services), benzylpenicillin (3 $\mu$g/ml) and streptomycin (7 $\mu$g/ml). The dishes were placed in a McIntosh and Fildes jar and gassed with humidified 95% $O_2$ and 5% $CO_2$. The medium was changed every 2 days.

*Analysis of tissue components.*—The cultured explants were fixed in Bouin's fluid and submitted to routine paraffin histology. Sections (7 $\mu$m) were stained with haematoxylin and eosin. The relative volumes of the 3 basic components (stroma, epithelium and space) were measured using a modification of the method of Chalkley (1943). The eyepiece graticule was divided into quadrants encompassing 25 randomly distributed points.

Using a Zeiss projection microscope, the image of section was projected on to a blank sheet of paper. The outlines of about 30 alveoli were drawn. Epithelium was classified into 3 types: squamous/cuboidal, columnar and stratified (Fig. 1). Using a map measurer, the total length of basement membrane occupied by each type of epithelium was measured and expressed as a percentage.

*Indices of proliferation.*—A 0·02% solution of vincristine sulphate (20 $\mu$l) was added to the culture medium. Between 2–3000 alveolar epithelial cells were counted and the number of metaphases recorded. Appropriate corrections for section thickness were used (Abercrombie, 1946; Philp and Buchanan, 1971).

Unless otherwise stated, 2 $\mu$Ci [$^{125}$I] UdR in 50 $\mu$l 0·9% sodium chloride solution was added to the culture medium 6 h before harvesting. Explants were then removed, blotted and weighed. Tissue was fixed in alcoholic Bouin's fluid for 24 h and washed 4 times in 70% alcohol to remove unbound isotope (Micklem, 1972; Pritchard and Micklem, 1972; Fidler, 1970). Samples were then counted in a Panax V-160 isotope counter.

### RESULTS

### *Morphological analysis: pilot studies*

Biopsy specimens of BPH and rat prostate were examined to determine the percentage volume occupied by epithelium, stroma and space. Each sample was scored using a total of 100 fields, which covered most of the section analysed. From each pool between 10 and 100 results were extracted at random with increments of 10 fields between successive group sizes. For each group of results the mean number of points over each tissue component was calculated. The standard error, as a percentage of the mean, decreased rapidly until about 40 fields had been scored, and this number of fields was used in all subsequent analyses.

The distribution of tissue components thoughout serial sections of 4 explants of BPH cultured for 2 days is shown in Fig. 2. Two of these explants were exposed to vincristine for the last 4 h of culture. Significant numbers of arrested cells were present throughout the depth of the tissue and not confined only to the surface region. There was, however, a drop in numbers towards the upper surface of the explants. In further studies, only sections from the middle of the explants were scored.

No metaphases were seen in either fresh or cultured BPH not exposed to vincristine.

### *Comparison of rat ventral prostate and BPH in culture*

The proportions of stroma, epithelium and space were determined. Using the same histological sections, alveolar epithelium was classified into 3 types and their relative proportions calculated. Results are summarized in the Table.

Regressive changes in rat prostate during culture were reflected by a fall in the percentage of columnar epithelial cells from 72 to 33% with a corresponding increase in the squamous/cuboidal and stratified epithelia, in the ratio 2 : 1. Nearly half of the alveoli in 4-day cultured

FIG. 1.—Epithelial types in BPH: A, Squamous; B, columnar; C, stratified ×600.

FIG. 2.—Chalkley analysis and metaphase counts from serial sections of BPH cultured for 2 days.

TABLE.—*Tissue Analysis of Explants of Rat Prostate and Human Benign Prostatic Hyperplasia*

| | % Tissue composition (±s.e.) | | | % Epithelial composition (±s.e.) | | |
|---|---|---|---|---|---|---|
| | Epithelium | Stroma | Space | Squamous/ Cuboidal | Columnar | Stratified |
| *Rat prostate** | | | | | | |
| Biopsy | 26·3±1·2 | 37·4±4·7 | 36·3±3·7 | 17·5±8·4 | 72·1±3·7 | 10·1±4·7 |
| Day 4 culture | 17·0±0·4 | 40·0±2·1 | 43·2±2·0 | 42·7±4·1 | 32·7±2·9 | 24·6±2·7 |
| *Benign prostatic hyperplasia*† | | | | | | |
| Biopsy | 11·8±0·9 | 69·5±1·4 | 18·4±0·7 | 17·2±3·3 | 52·8±4·0 | 24·6±2·9 |
| Day 2 culture | 10·2±0·5 | 77·8±1·0 | 12·0±0·7 | 16·5±2·5 | 35·7±2·5 | 45·4±1·5 |
| Day 4 culture | 11·1±0·5 | 76·1±0·9 | 12·7±0·7 | 14·5±2·4 | 36·6±2·5 | 48·5±1·6 |

*3 animals; 12 explants/treatment. †15 specimens; 4 explants/specimen/treatment.

rat prostate were of the squamous/cuboidal variety.

A different pattern was seen for BPH. Stratified epithelium became the dominant cell type in the cultured tissues. The proportion of squamous/cuboidal epithelium was not significantly changed. Neither the Chalkley nor the epithelial analysis revealed any significant difference between explants of BPH cultured for 2 and 4 days.

*Effects of culture on the metaphase incidence*

Explants of BPH were cultured for 2 days and removed at 2, 4 and 6 h after vincristine administration. The number

FIG. 3.—*In vivo* metaphase accumulation in rat ventral prostate following administration of vincristine (1 mg/kg body weight).

of metaphases/$10^3$ cells were respectively: 0·66±0·23; 6·18±0·8; 10·6±2·3 ($n=6$ using material from one tumour). The number of metaphases increased linearly with time after a lag period of 1·7 h.

Explants from 8 tumours were cultured for 2 and 4 days, and exposed to vincristine for the last 4 h of culture. The mean number of metaphases/$10^3$ cells/h on Day 4 (2·76±0·19) was approximately double that on Day 2 (1·40±0·32), indicating increased proliferative activity with time in culture.

Rat prostate was used in order to compare the mitotic activity *in vivo* with that after 4 days in culture. The cell production rate *in vivo* was calculated from the regression line of the number of cells entering metaphase/$10^3$ cells for animals exposed to vincristine for 2–6 h (Fig. 3). The metaphase accumulation was 0·6/$10^3$ cells/h, equivalent to a turnover time of 67 days.

Fewer metaphases were seen in cultured rat prostate. The average metaphase accumulation in tissue cultured for 4 days was 0·45/$10^3$ cells/h (mean value for 3 animals), corresponding to a turnover time of 93 days.

*[$^{125}$]I UdR incorporation*

BPH was cultured for 2 days and [$^{125}$I] UdR was added for the last 2–8 h of culture. The radioactivity in the explants was determined after elution of the unbound isotope with alcohol. There was a linear relationship between the concentration of the residual [$^{125}$I] in BPH and time of exposure to iododeoxyuridine (Fig. 4). The incorporation of [$^{125}$I] iododeoxyuridine into rat prostate was also linear with respect to time of incubation (Fig 5).

The incorporation of [$^{125}$I] tended to be slightly greater when the tissues were cultured in the presence of calf serum.

In all subsequent experiments, explants were exposed to [$^{125}$I] iododeoxyuridine for 6 h before harvesting.

Incorporation of [$^{125}$I] iododeoxyuridine was investigated in 6 benign specimens cultured for 0–8 days (Fig. 6). Incorporation was low on Day 0, initially increased with time in culture, then declined. In 4 of the 6 specimens of BPH, uptake reached a maximum on Day 4; with the remaining 2 there was a less pronounced peak on Day 6.

Despite the fact that 9 replicates were

FIG. 4.—Concentration of radioactivity in BPH following treatment with [$^{125}$I] UdR after 2 days in culture. Closed and open circles refer to material cultured with and without calf serum (10% v/v) respectively.

FIG. 5.—Concentration of radioactivity in rat ventral prostate following treatment with [$^{123}$I] UdR after 2 days in culture. Closed and open circles refer to material cultured with and without calf serum (10% v/v) respectively.

FIG. 6.—Uptake of [$^{125}$I] UdR by 6 BPH specimens for 0–8 days. Explants were exposed to [$^{125}$I] UdR for the last 6 h of culture. Each point is the mean ($\pm$s.e.) for 9 replicates.

FIG. 7.—Relationship between percentage epithelium and uptake of [$^{125}$I] UdR in BPH cultured for 2 days (open circles) and 4 days (closed circles) respectively. Mean values ($\pm$s.e.) are for Chalkley analysis of 3 sections from each explant.

used per determination in the above experiments, the standard error was in some cases quite large. This may well reflect marked variation in the cellular composition of the explants within each treatment group. In such cases, a highly significant correlation between the concentration of [$^{125}$I] and the percentage epithelium in the explants could be demonstrated and this allowed a precise estimate to be made of the increase in DNA synthetic activity during culture. Comparison of the slopes of the lines for 2 and 4 day cultured material (Fig. 7), showed that uptake [$^{125}$I] was 2·4 times greater on Day 4.

Addition of calf serum resulted in increased uptake without altering the overall pattern of incorporation (Fig. 8).

In contrast to BPH, rat prostate showed a gradual decrease in uptake of [$^{125}$I] UdR over the period of 2–8 days in culture: Day 2, 20·9±3·7; Day 4, 15·6±3·0; Day 6, 16·2±5·4; Day 8, 8·9±2·9 (mean values±s.e. for d/min per mg wet weight; $n$=9).

DISCUSSION

The results for the quantitation of the alveolar epithelium in fresh and cultured BPH reflected the morphological changes described previously by Harbitz (1973), McMahon and Thomas (1973) and McRae *et al.* (1973). There was some decrease in the size of the gland lumina in the cultured explants. The epithelium generally retained its *in vivo* appearance, but metaplasia and epithelial outgrowth were seen in acini transected at the medium/explant surface. Thus there was a significant increase in the proportion of stratified epithelium during the first 2 days of culture, mainly at the expense of columnar cells. In rat prostate, cultured under essentially the same conditions, the change from columnar to squamous/cuboidal epithelium was more striking. These findings support the impression that BPH is less sensitive than rat prostate to withdrawal of hormone support and the changes which occur during culture of BPH are more typical of a repair mech-

FIG. 8.—Effect of serum on the uptake of [$^{125}$I] UdR in cultured BPH. Mean values (±s.e.) are for 9 replicates using explants from a single tumour.

anism to injury (McMahon and Thomas, 1973) than of a castration effect.

Although morphological changes in BPH did not significantly progress after 2 days in culture, there was a pronounced increase in DNA synthesis and cell division between Days 2 and 4.

The cell kinetics of the cultured explants was investigated using the metaphase arrest agent vincristine, which has been shown to have advantages over Colcemid and vinblastine for *in vitro* studies (Riches, Littlewood and Thomas, 1972). An accurate estimate of the cell production rate requires a linear increase in the number of metaphases/$10^3$ cells with time and must be measured from the regression line (Smith, Thomas and Riches, 1974). Although this could be demonstrated in earlier studies with murine adenocarcinoma in culture (Riches, *et al.*, 1972), the number of metaphases seen in cultured explants of BPH was relatively small and it was difficult to test for linearity. The lag phase of 1·7 h suggests that diffusion of the arrest agent may be relatively slow in this type of tissue, and may explain why Harbitz (1973) noted metaphase accumulation in only one of 4 experiments in which BPH was exposed to Colcemid for 2 h after 2 and 4 days of culture. Thus the data given in the present work, based on 4 h exposure to vincristine, probably under estimate the cell production rate of cultured BPH. However, the 4 h accumulation of metaphases provides a useful comparative index of proliferative activity between the various treatments and demonstrates greater cell division on Day 4 than on Day 2 of culture.

The observed increase in proliferative activity during culture correlated well with the DNA synthetic activity as measured by the uptake of labelled iododeoxyuridine. [$^{325}$I] UdR is specifically incorporated into DNA (Hughes *et al.*, 1964) and has provided a useful approach to measurements of cellular proliferation and cell loss (Dethlefsen, 1974; Micklem, 1972) as the problems of reutilization inherent with [$^3$H]-thymidine are reduced (Dethlefsen, 1971, 1974; Clifton and Cooper 1973). It has an additional advantage over [$^3$H]-thymidine in that uptake of radioactivity can be coupled to an examination of the morphological features of the explant without the necessity of using autoradiographic techniques. This advantage has been used to demonstrate a highly significant correlation between uptake of [$^{125}$I]-iododeoxyuridine and percentage epithelium in the explants.

This approach also indicated activity on Day 4 was double than on Day 2, and that the uptake of labelled iododeoxyuridine was decreased after longer periods of culture. The overall pattern of uptake with time is consistent with that noted by McMahon and Thomas (1973) for the incorporation of [$^3$H]-thymidine into DNA and by Harbitz (1973) using an autoradiographic technique.

Thus, as a consequence of culture there there was a spontaneous and transient increase in proliferative indices. This pattern was not influenced markedly by the presence or absence of serum.

Stimulatory effects, as a result of culture, have also been reported for other sex hormone dependent tissues. Mueller, Herranen and Jervell (1958) and Russell and Thomas (1974) have noted enhancement, in the absence of oestrogen, of several metabolic parameters in cultured uterus. Kahn (1954) observed keratinization of mouse vaginal epithelium in culture. Thus, the possibility that the culture conditions may spontaneously lead to effects reminiscent of those normally expected under hormone stimulation is an added complication in assessing the value of organ culture in the study of hormone dependent tissues.

Morley, Wright and Appleton (1973) suggest that variations in proliferation indices, as a result of androgen stimulation in the castrate mouse, are more likely to be the result of changes in the proportion of proliferating and non-proliferating compartments ($G_0$ population), rather than due to a direct effect on the cell cycle time. Similar changes may be occurring in organ culture without androgen stimulation and this spontaneous wave of proliferative activity may well mask an androgenic effect during the period when cell division is most active.

In contrast to the results for BPH, rat prostate did not show an enhancement of DNA synthesis and cell division during culture. It has yet to be determined whether this represents an inherent difference between the two tissues or if it reflects an ageing phenomena. Simnett and Morley (1967) have examined the metaphase accumulation in the coagulating gland of mice of various ages, both *in vivo* and during organ culture. They found that tissue from young mice (aged 3 weeks) had a high metaphase accumulation *in vivo*, but that it fell during culture, presumably due to the absence of androgen to maintain proliferative activity. In material from older animals (up to 44 weeks), however, the metaphase accumulation was low *in vivo* and markedly increased during culture. They suggest that the increased proliferative activity *in vitro* appears to be due to isolation of the tissue from organizational influences which inhibit growth *in vivo*, rather than to dissection trauma. Thus, the balance between stimulating and inhibitory factors may well differ in the young and old animal and this in turn reflects changes seen in organ culture where systemic factors are removed.

Techniques for defining the proliferative capacity of human tumours are difficult to apply *in vivo*, although some results are available for BPH. Liavag (1968) measured the metaphase index following Colcemid administration $4\frac{1}{2}$ h before surgery and found an average of 94 metaphases/10 g tissue. On the assumption that the number of cells/g is of the order of $10^9$, it is evident that the cultured tissue showed a considerably higher proliferative activity than that seen *in vivo*. In this respect BPH would appear to be similar to prostate from older mice. In the latter case the degree of stimulation due to culture was about 110-fold (Simnett and Morley, 1967).

We are grateful to Mr B. H. Price for the supply of human prostatic specimens and to the Cancer Research Campaign for a grant in support of this work.

### REFERENCES

ABERCROMBIE, N. (1946) Estimation of Nuclear Population from Microtome Sections. *Anat. Rec.*, **94**, 239.

BAULIEU, E. E., LASNITZKI, I. &. ROBEL, P (1968) Metabolism of Testosterone and Action of Metabolites on Prostate Glands Grown in Organ Culture. *Nature, Lond.*, **219**, 1155.

CHALKLEY, H. W. (1943) Method of Quantitative Morphologic Analysis of Tissues. *J. natn. Cancer Inst.*, **4**, 47.

CLIFTON, K. M. & COOPER, J. M. (1973) Reutilization of Thymidine and Iododeoxyuridine by Mouse Mammary Carcinoma Strain MTG–B. *Proc. Soc. exp. Med.*, **144**, 1145.

DETHLEFSEN, L. A. (1971) An Evaluation of Radioiodine-labelled 5-iodo-2-deoxyuridine as a Tracer for measuring Cell Loss from Solid Tumours. *Cell tissue Kinet.*, **4**, 123.

DETHLEFSEN, L. A. (1974). $^3$H-5-iodo-2′-deoxyuridine Toxity. Problems in Cell Proliferation Studies. *Cell tissue Kinet.*, **7**, 213.

FIDLER, I. J. (1970) Metastasis: Quantitative Analysis of Distribution and Fate of Tumor Emboli Labelled with $^{125}$I-5-iodo-2$^1$-deoxyuridine. *J. natn. Cancer Inst.*, **45**, 773.

FULLER, D. J., COLESHILL, S. M., CHAN, K. M. B. & THOMAS, G. H. (1974) Insulin and Testosterone Responses in Cultured Prostate. *J. Endocr.*, **63**, 59.

FRANKS, L. M., RIDDLE, P. M., CARBONELL, A. W. & GEY, G. O. (1970) A Comparative Study of the Ultrastructure and Lack of Growth Capacity of Adult Human Prostate Epithelium Mechanically Separated from its Stroma. *J. Path.*, **100**, 113.

HARBITZ, B. T. (1973) Organ Culture of Benign Nodular Hyperplasia of Human Prostate in Chemically Defined Medium. *Scand. J. Urol.*, **7**, 6.

HUGHES, W. L., COMMERFORD, S. L., GITLIN, D., KRUEGER, R. C., SCHULTZE, B., SHAH, V. & REILLY. P. (1964) Deoxyribonucleic Acid Metabolism *in vivo*: I. Cell Proliferation and Death as Measured by Incorporation and Elimination of $^{125}$I UdR *Fedn Proc.*, **23**, 640.

KAHN, R. H. (1954) Effect of Oestrogen and of Vitamin A on Vaginal Cornification in Tissue Culture. *Nature, Lond.*, **174**, 317.

LASNITZKI, I. (1965) Action and Interaction of Hormones and 3-methylcholanthrene on the Ventral Prostate Gland of the Rat *in vitro*: I Testosterone and Methylcholanthrene. *J. natn. Cancer Inst.*, **35**, 339.

LIAVAG, I. (1968) Mitotic Activity of Prostatic Epithelium. A Study by Means of Colcemid. *Acta path. microbiol. scand.*, **73**, 19.

LOSTROH, A. J. (1968) Regulation by Testosterone and Insulin of Citrate Secretion and Protein Synthesis in Explanted Mouse Prostates. *Proc. natn. Acad. Sci. U.S.A.*, **60**, 1312.

McMAHON, M. J., BUTLER, A. V. J. & THOMAS, G. H. (1972) Morphological Responses of Prostatic Carcinoma to Testosterone in Organ Culture. *Br. J. Cancer*, **26**, 388.

McMAHON, M. J. & THOMAS, G. H. (1973) Morphological Changes of Benign Prostatic Hyperplasia in Culture. *Br. J. Cancer*, **27**, 323.

McRAE. C. U., GHANDADIAN, R., FOTHERBY, K. & CHISHOLM, G. D. (1973) The Effect of Testosterone on the Human Prostate in Organ Culture. *Br. J. Urol.*, **45**, 156.

MICKLEM, H. S. (1972) Cell Proliferation in Haematopoietic Spleen Colonies of Mice: Difference between Colonies Derived from Injected Adult Bone Marrow and Foetal Liver Cells. *Cell. tissue Kinet.*, **5**, 159.

MORLEY, A. R., WRIGHT, N. A. & APPLETON, D. (1973) Cell Proliferation in the Castrate Mouse Seminal Vesicle in Response to Testosterone Propionate. *Cell tissue Kinet.*, **6**, 239.

MUELLER, G. C., HERRANEN, A, M. & JERVELL, K. F. (1958) Studies on the Mechanism of Action of Estrogens. *Rec. Prog. horm. Res.*, **14**, 95.

PHILP, J. R. & BUCHANAN, T. J. (1971) Quantitative Measurements on Finite Tissue Sections. *J. Anat.*, **108**, 89.

PRITCHARD, N. & MICKLEM, H. S. (1972) Immune Responses in Congenitally Thymus-less Mice. I. Absence of Response to Oxazolone. *Clin. & exp. Immunol.*, **10**, 151

RICHES, A. C., LITTLEWOOD, V. & THOMAS, D. B. (1972) The Growth Potential of Tumour Explants. *J. Anat.*, **114**, 299.

RUSSELL, S. L. & THOMAS, G. H. (1974) Oestradiol Uptake and Retention, and High-affinity Binding Sites in Cultured Rabbit Uterus. *Biochem. J.*, **144**, 99.

SANTTI, R. S. & JOHANSSON, R. (1973) Some Biochemical Effects of Insulin and Steroid Hormones on the Rat Prostate in Organ Culture. *Expl cell. Res.*, **77**, 111.

SIMNETT, J. D. & MORLEY, A. R. (1967) Factors Controlling Growth of Prostatic Epithelium. A Comparision of Mitotic Activity in Mice of Different ages *in vivo* and in Organ Culture. *Expl cell Res.*, **46**, 29.

SMITH, S. R., THOMAS, D. B. & RICHES, A. C. (1974) Cell Production in Tumour Isografts Measured using Vincristine and Colcemid. *Cell tissue Kinet.*, **7**, 529.